# Reform in Tuberculosis Work

**Published:** 1917-01-27

**Authors:** 


### REFORM IN TUBERCULOSIS WORK.

### III. The Tuberculosis Officer.*

By A TUBERCULOSIS WORKER.

It is a fact predictable enough from the constitution of human nature that every nation ascribes to itself too much share in any innovation or discovery reckoned creditable, and too little in any reckoned discreditable. To take an instance of the former kind from the field of medicine, we in England say that Harvey was the sole discoverer of the circulation of the blood. But in Lanciani's impressive work on physiology Italian names are also prominently mentioned. On the other hand an anti-conceptional appliance used to be called English in France, and French in England. It is therefore well to remember always the proverb about self-praise, and to accept that judgment only which is commonly cited in the words *securus judicat orbis terrarum*. Applying this great rule to our present little subject, one finds that on the Continent France is reckoned the home of the tuberculosis dispensary movement. Again, in Germany there have been established not only the *Fürsorge* for tubercle, but also, sometimes combined with it in the same building, that for venereal disease, and even that for alcoholism. It is no discredit, then, to Sir Robert Philip's energy and practical spirit if one begins by saying that criticism of the tuberculosis officer and his institution, in this country, cannot be deprecated as premature, as carping at pioneer work.

### The Selection of Tuberculosis Officials.

The great extension of public anti-tuberculosis activity in the United Kingdom which followed upon the late Dr. Bulstrode's excellent report, and upon the passing of the National Insurance Act, came so comparatively suddenly as to find a scarcity of medical men having practical acquaintance with the subject. That scarcity could not be helped, but its effect was needlessly aggravated by the action of local elective bodies in frequently ignoring, in practice, tuberculosis experience as a qualification. Professor Osler pointed out not long ago the need of some central control of local anti-tuberculosis work, and certainly facts of common experience bear out his demand. Here is a case in point. Two gentlemen, one newly qualified, the other a general practitioner in the 'forties, married, and with an adolescent family, become school medical inspectors to a municipality. This body in the course of a year or two takes up tuberculosis dispensary work and advertises for tuberculosis officers. The two gentlemen mentioned, obeying a natural impulse (for the salary offered in the new posts is about £100 a year more than they are getting), set about obtaining these appointments. Although devoid of tuberculosis experience, they do obtain them, and what is more, move on in the course of another year to better-paid tuberculosis appointments, defeating medical men of ten years' exclusive tuberculosis work, better qualified generally, and of much greater special clinical competence, competence gained, too, on very exiguous remuneration. The injustice of this sort of thing, which has happened all over the country, may be relied upon to breed cynicism, disaffection, ignorance, and slackness. Professor Osler's call for central control is certainly justified.

### Propinquity and Prejudice.

Not that one would use any forcible epithets by which to describe such action of local authorities. Like much conduct upon which indignation is expended, it probably all came about very easily and even naturally. These public health committees are composed of ordinary men; and in any assembly, however small, and even of extraordinary men, the emotional tone is raised, as compared with that of the individual, and the intellectual tone similarly lowered. Hence a local candidate (a graduate of the local university, say) attracts subconscious support. Those, too, who are initiating a new move-

* Previous articles appeared January 13 and 20.

ment upon which they are not closely informed, desire knowledge indeed, but not from subordinates. They are like the rector unanxious for a curate who preaches too good a sermon; the instinct of self-preservation comes unconsciously into play. Out of three candidates for the post of resident to a newly started sanatorium in the provinces, the victor was the only one devoid of previous sanatorium experience; he was also in delicate health. But then he had been house physician to one of the local medical *cognoscenti*, and in addition to the extra opportunities of canvassing which this gave him, it gave also to his superiors the assurance that he could be relied upon to keep his place and give himself no airs of knowledge. *Medicus medicum odit*: these subconscious prejudices fail not of their effect, and what is needed is disinterested control to neutralise them.

The Fallacy of Oral Dosage with Tuberculin.

Another phenomenon somewhat attributable to such elections is the low level of clinical proficiency obtaining as yet amongst tuberculosis officers. German experiments, well confirmed some years ago by Dr. Radcliffe in the laboratory at the King's Sanatorium, Midhurst, under Professor Bullock's inspiration, have made it as sure as anything can be sure therapeutically, that tuberculin given by the mouth is without effect. No manifestation of *Allergie* is obtainable after oral administration; huge doses of tuberculin given without any previous administration produce no reaction, proving as innocuous as a drink of saline solution. Oral administration of tuberculin is, in short, waste of tuberculin (and some kinds are very dear just now). This finding has been nearly universally accepted by real tuberculosis clinicians, so much so that the fact that Dr. Latham described a modification of the opsonic index following a mouth-dose of tuberculin has now helped to discredit that chameleon-like thing as a practical clinical guide. Yet it is quite common to see tuberculosis officers giving children tuberculin out of a teaspoon. Again, in the first number of Professor Forlanini's international journal on the artificial pneumothorax treatment of consumption, the list of subscribers from England, eleven in number, contains only one name of a tuberculosis officer, and he a subordinate one. The idea that a tuberculosis officer's work is purely sanitary is negatived by the very first of his duties, that of early diagnosis of phthisis, which calls for the closest and most persevering clinical study. Many are diagnosing the disease on the strength of tuberculin reactions long exploded.

The writer once heard at a conference of tuberculosis officers a gentleman with no other tuberculosis experience put forward a "modest proposal" for lessening overcrowding in an industrial district by simply passing a by-law against taking lodgers. As though vastly less *doctrinaire* enactments have scarcely emerged as yet from a dead-letter stage! The conservative practitioner finds it easy to evade the notification of births regulations; many refuse to notify some of their cases of tuberculosis. As far back as Mr. John Burns's time, a by-law was passed forbidding the use of "cut-outs" on motor-cycles; and every summer since, after a short initial silence, the roads have resounded to machine-gunlike reports just the same. Housing reform and all the other great problems of social amelioration follow on many other grounds besides that of tuberculosis prophylaxis, and such partial solutions of them as will be practicable for some time have long had a prominent place in the programme of every sanitary authority. There is no need of more fuglemen for this cause; but there is a great need in every district for good clinical work amongst the tuberculous.

Surgical Tuberculosis.

Under the National Insurance Act, resident institutional treatment is provided for suitable cases of consumption, but there is none for surgical tubercle: such cases must rely as before on the voluntary special and general hospitals, admission to which is difficult for many reasons, such as lack of accommodation and consequent delay, or lack of means on the part of the patient's friends. At the present time it is most especially difficult, because of the number of surgical beds (far too few in peace-time) occupied everywhere by wounded soldiers, and of the heavy demands on the time of the diminished number of hospital surgeons. Now a very common class of case at tuberculosis dispensaries is children suffering from tuberculous cervical glands; and in place of year-long tuberculin treatment, with perhaps no very visible good result, it might be better at dispensaries—and there are many such—as are provided with a bed or two, that the necessary excision should be performed by the tuberculosis officer himself. There is also a great call for conservative treatment of bone and joint tuberculosis in children, at all events preliminary treatment, to be undertaken at the patient's home. With the advantage of open-air shelters, the provision of which is already widely authorised, a great deal can be done towards preventing deformity and initiating a cure. Courses of study at institutions like the Cripples' Home at Alton would be of the greatest advantage to tuberculosis officers.

Such, then, are some of the real defects, those most noticeable in detailed experience, of our public anti-tuberculosis campaign. It must not be thought that in war-time, and such a war-time, they recede in importance, for war itself is a potent cause of the disease, both in the combatants themselves and, through privation, in the home population. It is significant that those countries which have been pecuniarily hardest hit by the war are also the loudest (*e.g.* Austria, and, through M. Maeterlinck just lately, Belgium) in their demands upon the public purse for further anti-tuberculosis measures in the near future. But if the aim, as in this country it has so largely been in the past, be merely to satisfy appearances and exercise patronage, why then there may ensue an awakening to facts even more disagreeable than those already experienced.

